# MANAGEMENT OF DEPRESSION IN OLDER ADULTS RECEIVING CARE IN MEDICAL SETTINGS

**DOI:** 10.1093/geroni/igac059.1089

**Published:** 2022-12-20

**Authors:** Cecilia Poon

**Affiliations:** Nebraska Medicine, Omaha, Nebraska, United States

## Abstract

Although rates of major depressive disorder are lower among older adults, depressive symptoms are a common presentation for aging individuals in medical settings. Unique challenges arise when treating depressive symptoms co-occurring with brain health concerns in older adults with a complex medical history. This presentation reviews how cognitive behavioral interventions for later-life depression are relevant for mental health practitioners who work in primary care and general medical settings. Specific clinical and multicultural considerations will be highlighted to support clinicians and interdisciplinary teams to work effectively with older adults who have co-existing depressive symptoms and cognitive concerns.

